# Supplementary material for: Genome-Wide Identification and Expression Analysis of the RADIALIS-like Gene Family in Camellia sinensis
Source: Plants (Basel). 2023 Aug 24;12(17):3039. doi: 10.3390/plants12173039 (PMC10490161; doi:10.3390/plants12173039)
Supplement: Supplementary file 1 [file plants-12-03039-s001.zip › Supplementary table S1.pdf]

Supplementary table S1. PCR primers used in the cloning of *CsaRLs*

Gene name	Forward primer (5′-3′)	Reverse primer (5′-3′)
CsaRL1a	F: ATGGCATCAATGTCCTCCCG	R: CTTCGACGGAGCTTCAAATATTTCA
CsaRL1b	F: ATGGCATCAATGTCCTCCC	R: ATTACTACTACTACCTGTGTAGTTTGGGAA
CsaRL3a	F: ATGGCGTCAAACTGCTCTTCC	R: GTTTGATCTGTAATTGGGAAATGGGA
CsaRL3b	F: TGGGTTCGAACTCATCAAATTGGAG	R: TTGCAGTTTCAGACACTTCAG
CsaRL3c	F: ATGGCATCAAGTTCTCTCAGTT	R: GTGCTGCTTAAGGAACCTCA
CsaRL4a\CsaRL4b	F: ATGGCATCGAACTCTTTCAGTTCTTC	R: GAGATAATTACCACCACTCTGCCTT
CsaRL4c	F: ATGGCATCAAGCTCTATGAAATCTT	R: CCAACCTCTACCATTGCTAGCA
