# Supplementary material for: Genome-Wide Identification and Expression Analysis of the RADIALIS-like Gene Family in Camellia sinensis
Source: Plants (Basel). 2023 Aug 24;12(17):3039. doi: 10.3390/plants12173039 (PMC10490161; doi:10.3390/plants12173039)
Supplement: Supplementary file 1 [file plants-12-03039-s001.zip › Supplementary table S2.pdf]

Supplementary table S2. Primers used for qRT-PCR

Gene name	Forward primer (5′-3′)	Reverse primer (5′-3′)
qPCR-CsaRL1a	F: TGGCTCTGGCAGTTGAA	R: CTAGTTCCTCCTGTAGTTTGGG
qPCR-CsaRL1b	F: GAAAAGGCTCTGGCTGTGTAT	R: AATTACTACTACTACCTGTGTAGTTTGG
qPCR-CsaRL3a	F: CTCATGTAGCTCCGGCTCT	R: GATCTGTAATTGGGAAATGGGACAC
qPCR-CsaRL3b	F: GAGAGGTGGGAGAACATTGCG	R: CATTGCAGTTTCAGACACTTCAG
qPCR-CsaRL3c	F: ATGGCATCAAGTTCTCTCAGTTCAC	R: TCAGTGCTGCTTAAGGAACC
qPCR-CsaRL4a\CsaRL4b	F: ATGGCATCGAACTCTTTCAG	R: TTAGAGATAATTACCACCACTCTGCC
qPCR-CsaRL4c	F: ATGGCATCAAGCTCTATGAAATC	R: TTACCAACCTCTACCATTGCTAGC
Csaβ-actin	F: GCCATCTTTGATTGGAATGG	R: GGTGCCACAACCTTGATCTT
