# Supplementary material for: Genome-Wide Identification and Expression Analysis of the RADIALIS-like Gene Family in Camellia sinensis
Source: Plants (Basel). 2023 Aug 24;12(17):3039. doi: 10.3390/plants12173039 (PMC10490161; doi:10.3390/plants12173039)
Supplement: Supplementary file 1 [file plants-12-03039-s001.zip › Supplementary table S3.pdf]

Supplementary table S3. Primers used for Yeast two-hybrid assay

Gene name	Forward primer (5′-3′)	Reverse primer (5′-3′)
pGADT7-CsaRL1a	F: CCGGAATTCATGGCATCAATGTCCTCCCG	R: CGCGGTACCCTTCGACGGAGCTTCAAATATTTCA
pGADT7-CsaRL1b	F: CCGGAATTCATGGCATCAATGTCCTCCC	R: CGCGGTACCATTACTACTACTACCTGTGTAGTTTGGGAA
pGADT7-CsaRL3a	F: CCGGAATTCATGGCGTCAAACTGCTCTTCC	R: CGCGGTACCGTTTGATCTGTAATTGGGAAATGGGA
pGADT7-CsaRL3b	F: CCGGAATTCTGGGTTCGAACTCATCAAATTGGAG	R: CGCGGTACCTTGCAGTTTCAGACACTTCAG
pGADT7-CsaRL3c	F: CCGGAATTCATGGCATCAAGTTCTCTCAGTT	R: CGCGGTACCGTGCTGCTTAAGGAACCTCA
pGADT7-CsaRL4a/pGADT7-CsaRL4b	F: CCGGAATTCATGGCATCGAACTCTTTCAGTTCTTC	R: CGCGGTACCGAGATAATTACCACCACTCTGCCTT
pGADT7-CsaRL4c	F: CCGGAATTCATGGCATCAAGCTCTATGAAATCTT	R: CGCGGTACCCCAACCTCTACCATTGCTAGCA
