# Supplementary figures and images for: Genome-Wide Identification and Expression Analysis of the RADIALIS-like Gene Family in Camellia sinensis
Source: Plants (Basel). 2023 Aug 24;12(17):3039. doi: 10.3390/plants12173039 (PMC10490161; doi:10.3390/plants12173039)

Supplementary figure S1: The expression pattern of *CsaRL* genes under abiotic stresses.

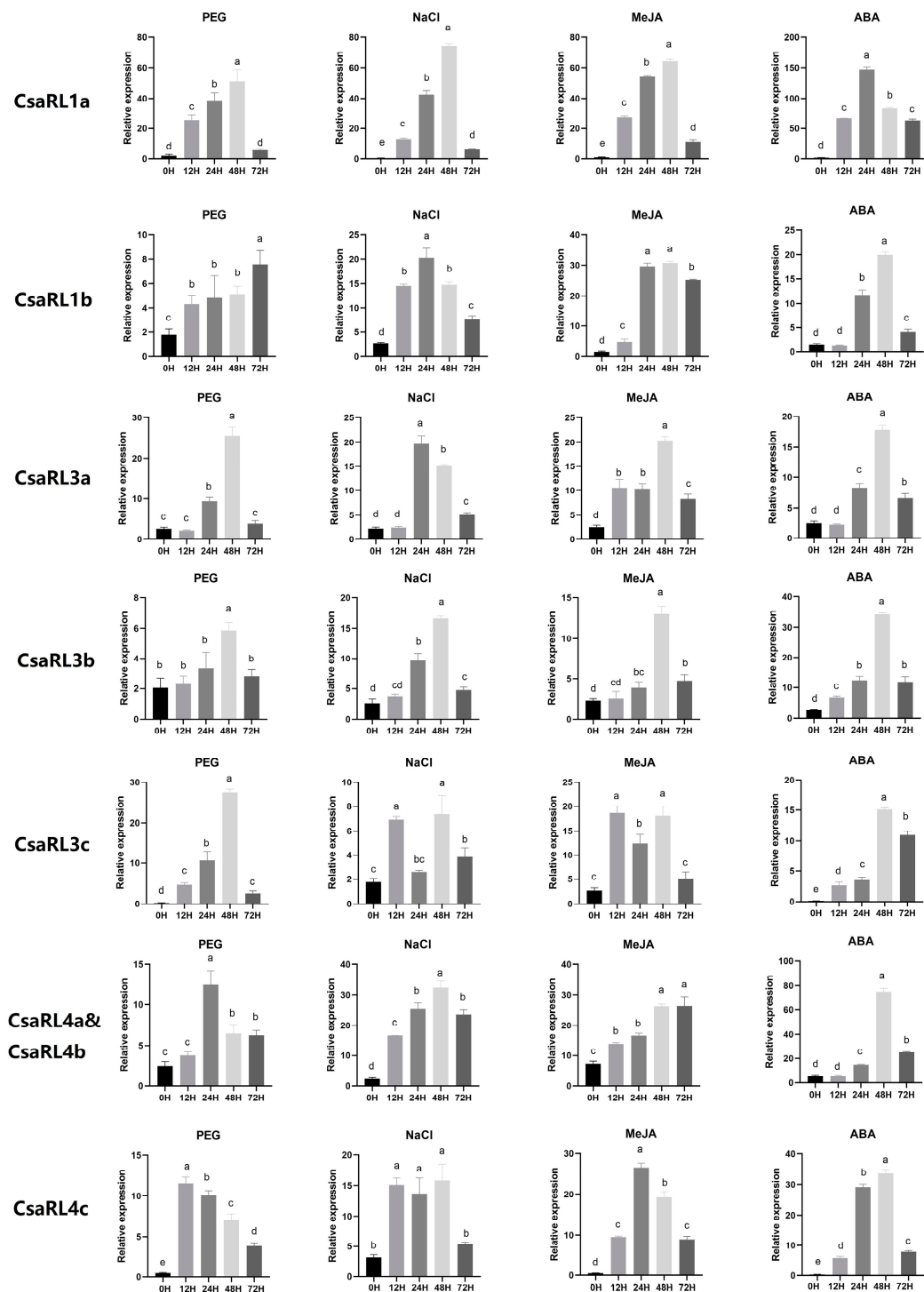

Supplement: Supplementary file 1 [file plants-12-03039-s001.zip › Supplementary figure S1.pdf]
